# Household income, active travel, and their interacting impact on body mass index in a sample of urban Canadians: a Bayesian spatial analysis

**DOI:** 10.1186/s12942-019-0168-x

**Published:** 2019-02-06

**Authors:** Hui Luan, Dana Ramsay, Daniel Fuller

**Affiliations:** 1 School of Geodesy and Geomatics, Wuhan University, Wuhan, 430079 Hubei China; 2 Department of Geography, University of Oregon, Eugene, OR 97403 USA; 3 School of Public Health, University of Saskatchewan, Saskatoon, SK Canada; 4 Canada Research Chair in Population Physical Activity, School of Human Kinetics and Recreation, Physical Education Building, Memorial University of Newfoundland, St. John’s, NF A1C 5S7 Canada

**Keywords:** Body mass index (BMI), Multi-level model, Active travel, Socioeconomic deprivation, Bayesian spatial modeling

## Abstract

**Background:**

Active travel for utilitarian purposes contributes to total physical activity and may help counter the obesity epidemic. However, the evidence linking active travel and individual-level body weight is equivocal. Statistical modeling that accounts for spatial autocorrelation and unmeasured spatial predictors has not yet used to explore whether the health benefits of active travel are shared equally across socioeconomic groups.

**Methods:**

Bayesian hierarchical models with spatial random effects were developed using travel survey data from Saskatoon, Canada (N = 4625). Differences in log-transformed body mass index (BMI) were estimated for levels of active travel use (vehicular travel only, mixed vehicular/active travel, and active travel only), household income, and neighbourhood deprivation after controlling for sociodemographic and physical activity variables. The modifying effect of household income on the association between active travel and BMI was also evaluated.

**Results:**

Significant and meaningful decreases in BMI were observed for mixed (β = − 0.02, CrI − 0.036 to − 0.004) and active only (β = − 0.043, CrI − 0.06 to − 0.025) compared to vehicular only travelers. BMI was significantly associated with levels of household income and neighbourhood deprivation. Accounting for the interaction between travel mode and household income, decreases in BMI were observed for active only compared to vehicular only travellers in the highest income category (β = − 0.061, CrI − 0.115 to − 0.007).

**Conclusion:**

Strategies to increase active travel use can support healthy weight loss and maintenance, but the opportunity to benefit from active travel use may be limited by low income. Considerations should be given to how interventions to increase active transportation might exacerbate social inequalities in BMI. Spatial statistical models are needed to account for unmeasured but spatially structured neighbourhood factors.

**Electronic supplementary material:**

The online version of this article (10.1186/s12942-019-0168-x) contains supplementary material, which is available to authorized users.

## Introduction

The increasing prevalence of obesity, defined by a body mass index (BMI) of 30 or higher, has been described as a public health challenge of global pandemic proportions [1]. In Canada, the prevalence of obesity has increased over the past three decades [2]. Approximately 18.3% of Canadian adults were considered obese in 2011, a percentage that is expected to rise to 21.2% across all provinces by 2019 [2]. The relationships between overweight and obesity and co-morbid health conditions are well documented [3]. The annual direct costs attributable to overweight and obesity in Canada were estimated to be between $4.6 billion and $7.1 billion, and is projected to rise to $8.8 billion by 2021 [4]. Interventions directed at obesity can improve the health status of Canadians while reducing direct costs to the healthcare system.

Regular physical activity is effective in the primary and secondary prevention obesity [5]. A nationally representative accelerometer study (2007–2009) indicates that only 15% of Canadian adults are sufficiently active to meet Canada’s physical activity recommendations, suggesting there is much room for improvement through initiatives that design, deliver, and prioritize healthy active living [6]. Transportation and urban planning researchers have examined physical activity from the perspective of active transportation, defined as any self-propelled, human-powered mode of transportation, such as walking or bicycling [7, 8]. Active transportation cycling and transit use contribute to total physical activity and contribute to total physical activity [8, 9].

In a systematic review of 30 studies examining the relationship between active transport and BMI and/or waist circumference at the individual level, less than half (13) reported associations in the expected direction (increased active transport leads to lower body weight) [10]. While the public health community is broadly supportive of policies to increase active travel, the limited evidence linking active travel to obesity reduction is in need of additional support [10, 11].

Socioeconomic status (SES) is another factor that is associated with BMI [12, 13]. Multi-level studies of urban Canadians indicate that individuals who live in socially [12] and materially [14] deprived neighborhoods have higher BMIs than their counterparts in more privileged neighborhoods. Limited access for low-income persons to the physical, material and psychosocial resources that support healthy weight maintenance remains an important obstacle to tackling the obesity problem [15–17]. For example, there is evidence that low SES areas may include more high speed roads [18, 19] and less active transportation infrastructure (e.g., cycle tracks) [20]. Given that low SES is associated with greater BMI and with poorer access to infrastructure that supports active transportation, it is plausible that active travel may not be associated with BMI among low SES groups [17] because the positive benefits of active transportation may be outweighed by socioeconomic status.

However, an important challenge in understanding associations between SES, active transportation, and BMI is statistical modeling. Often, random effects modeling is used to include area-level confounders to help explain the impact of active transport on BMI, adjusting for individual-level characteristics such as gender and age. For example, Scott et al. [21] applied two-level non-spatial linear models to explore the extent to which area-level socioeconomic status affect walking and BMI by race. To the best of our knowledge however, no previous studies used spatial statistical modeling to analyze the relationship between SES, active transportation and BMI. Not using spatial statistical models fails to account for unmeasured, potentially important, and spatially structured area-level confounders. As previously discussed, SES areas may have more high speed roads and less cycling infrastructure that hinder active transportation. Also, it is not possible or practical to collect all potential area-level confounders of BMI. Using spatial random effects as a proxy for these unmeasured area-level confounders [22] can provide more reliable statistical inferences.

Accounting for individual-, household-, and area-level confounders, the key objective of this study is to apply multi-level spatial statistical models to explore the relationship between SES, active transportation, and BMI, with three sub-objectives. First, to examine the association between self-reported travel mode and BMI in a sample of Canadian adults living in an urban center. Second, to examine the potential modifying effects of household income on the relationship between travel mode and BMI, given that income may limit the opportunity to benefit from physical activity achieved via active transportation. Third, to compare the results between spatial and non-spatial statistical modeling.

## Methods

This observational study used cross-sectional survey data from the 2013 Saskatoon Household Travel Survey (SHTS) [23]. The survey collected information on individual travel behaviors from a stratified random sample of households in the Saskatoon CMA from September 5th to October 31st, 2013. Participants provided verbal informed consent to participate. Pre-selected households were recruited via notification letter and/or telephone call, and assigned a trip diary date between Monday and Thursday; a total of 3595 households completed the survey. In a second step, the SHTS data were linked to 2006 Census data for the 336 Dissemination Areas (DA) within Saskatoon. DAs are the smallest geographic area for which Census data are disseminated, and are comprised of one or more neighboring city blocks representing a population ranging from 400 to 700 [24]. The linked, multilevel dataset thus features individual-level data nested within household and DA (area-level) units. Ethical approval was obtained from the University of Saskatchewan ethics office.

### Dependent variable

#### BMI

Using the same measure as the Canadian Community Health Survey, self-reported height and weight data were obtained from the SHTS for each individual in participating households [25]. The weight in kilograms was divided by the height in meters squared (kg/m^2^) to obtain BMI. BMI was log-transformed and modeled as a continuous outcome in order to examine the effect of confounders on absolute differences in BMI.

### Independent variables

#### Individual and household demographics

Self-reported sociodemographic information was obtained from the SHTS. Potential confounders were identified a priori on the basis of a literature scan, including a recent review paper which summarized the confounders adjusted in previous similar studies [10]. Individual-level characteristics included age and sex. Persons less than 19 years of age were excluded from the analysis (N = 1092), given that BMI is calculated differently for children and teens than for adults [26]. Age in years was recoded into categories (19–34 years, 35–49 years, 50–64 years, and ≥ 65 years) consistent with methods used in the Canadian Census [27].

Household-level characteristics included household income and the presence/absence of young children (< 5 years) in the home. Household income was recoded from six to four categories for simplicity and to highlight disparities in financial resources (< $25,000, $25,000–49,999, $50,000–74,999, and ≥ $75,000), consistent with the method used in the Canadian Community Health Survey [28]. Categorization was informed by median after-tax income figures, which range from $25,800 for unattached individuals to $68,000 for economic families in Canada [29].

#### Active transport and leisure time physical activity

The SHTS trip diary captured the self-reported mode of travel for all trips made during a single weekday [23]. Active travel was derived by measuring the number of active trips (on foot, bicycle or transit) as a proportion of the total number of trips made using all modes. Persons who made zero trips on the trip diary date were excluded from the analysis (N = 1990). These people were removed because without trips we could not assign them to a transportation mode category. Transportation mode was recoded into three meaningful categories: vehicular travel only (all trips using motor vehicle), mixed vehicular/active travel (any combination of motor vehicle and active trips), and active transportation only (all trips using public transit, walking, and cycling).

In order to measure the independent effect of travel mode on BMI, the analysis controlled for leisure time physical activity using a validated, single-item physical activity measure [30].

#### Area-level confounders

**Fig. 1:**
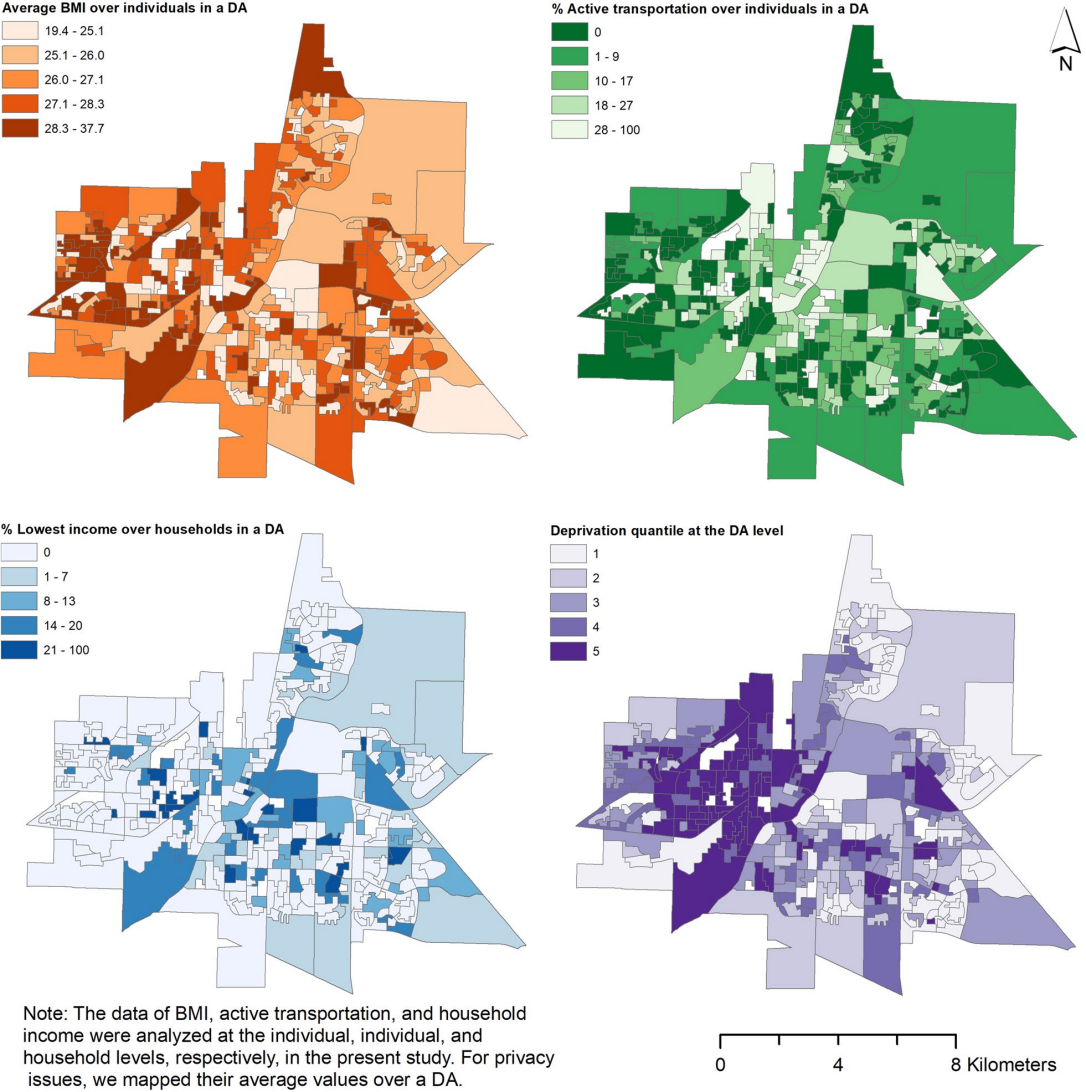
Maps of BMI, % of active transportation,  % of lowest household income, and deprivation in Saskatoon at the DA level

Measures of urban form and area-level deprivation, defined as a state of observable and demonstrable income and social disadvantage relative to the local community [31], were derived for each DA from Census data. Deprivation is an index developed by the *Institut National de Santé Publique du Québec* (INSPQ) that combines dimensions of material and social deprivation at the DA Census unit [31]. Principal component analysis was used to integrate socioeconomic indicators into the two-component factor structure. Material deprivation is comprised of three factors that include the proportion of people aged 15 years and older without a high school diploma; the employment to population ratio of people aged 15 years and older; and the average income of individuals 15 years and older. Social deprivation is comprised of three factors that include the proportion of individuals aged 15 years and older living alone; the proportion of individuals aged 15 years and older who are separated, widowed or divorced; and the proportion of single-parent families. Deprivation is measured in quintiles, where Q1 and Q5 are the least and most deprived populations, respectively [31]. Figure 1 shows the spatial distribution of BMI, % of active transportation, % of lowest household income, and deprivation in Saskatoon at the DA level. It should be noted that we averaged BMI, active transportation, and lowest household income at the DA level due to privacy issues, although they were analyzed at the individual, individual, and household levels, respectively. At the DA level, the spatial patterns of average BMI and % of active transportation seem align with each other. A cluster of highest deprivation located toward the western side of the city.

Continuous urban form variables included population density (persons/km^2^), an index of Canadian Active Living Environments (Can-ALE, often referred to as “walkability”) [32], and number of roads within a DA measured by calculating the centroid of each road and counting points in polygons. Road centroid number is a proxy for street connectivity and/or accessibility, a feature which is captured several ways in the built environment literature [33–35]. Research suggests that density, walkability, and street connectivity promote transit ridership and other forms of active transportation [34–36].

### Statistical analysis

A three-level hierarchical model was developed to analyze the data. The model was implemented within the Bayesian framework using WinBUGS1.4 [37]. To achieve better normality, we log-transformed participants’ BMI values. The multi-level model for an individual *i* in household *j* at DA *k* can be written as: $Y_{ijk} = \alpha + \beta *X_{ijk} + \beta^{\prime } *X_{jk}^{\prime } + \beta^{{\prime \prime }} *X_{k}^{{\prime \prime }} + \varepsilon_{ijk} + \varepsilon_{jk}^{\prime } + \varepsilon_{k}^{{\prime \prime }}$, where Y_ijk_ is the log-transformed BMI value, and X_ijk_, $X_{jk}^{\prime }$, and $X_{k}^{{\prime \prime }}$ are the independent variables at the three levels with corresponding coefficients $\beta , \, \beta^{{\prime }}$ and $\beta^{{\prime \prime }}$, respectively. In Bayesian analysis, the significance of independent variables is determined by their coefficients’ 95% Credible Interval (CrI), which is the range within which there is a 95% probability that the posterior mean occurs. An independent variable is significantly associated with the outcome when its coefficient’s 95% CrI does not cover zero. Three random effects $\varepsilon_{ijk}$, $\varepsilon_{jk}^{\prime }$, and $\varepsilon_{k}^{{\prime \prime }}$ were incorporated in the model to account for variability and unmeasured confounders at the individual, household, and DA level, respectively. We used non-spatial and spatial distributions, respectively, to model $\varepsilon_{k}^{{\prime \prime }}$ in two seprate mdoels.^1^ The former (Model 1) is a random noise term while the latter (Model 2) is a spatial random effect term, which ‘borrows’ information from neighboring DAs and acts as a proxy of unmeasured and spatially structured area-level confounders [22, 38]. Neighbors were defined as areas sharing at least one vertex, an approach most commonly used in the literature which remains a good choice for spatial smoothing compared with other adjacency definitions based on distances or covariate similarities [39].

To explore whether the household income and travel modes have an interacted impact on BMI, we fitted two more models with an interaction term, household income*travel mode (Models 3 and 4 representing models with spatial and non-spatial random effects, respectively). Models were compared using Deviance Information Criterion (DIC). A better model is the one with a lower DIC value [40]. More details of model specification are provided in the Additional file 1: Appendix.

## Results

### Sample characteristics

Attributable to the missing covariate issue, 801 individuals were excluded from the sampling data (N = 5426) for the analysis. The majority had missing data for household income (N = 653). Analysis was limited to 4625 observations (nested in in 2726 households at 330 DAs) with no missing data. Table 1 shows the descriptive statistics for BMI and the distribution of observations by travel mode for categorical confounders. Mean BMI was greater for those who used vehicular travel only (26.95, SD 5.16) and lowest (25.32, SD 4.93) for those who used active travel only. Mean BMI was greater for older adults, males, and less physically active individuals [2]. Mean BMI was higher with more neighborhood deprivation. Younger adults (< 35 years), persons of low income, and individuals from deprived neighborhoods use active travel modes more than their respective counterparts. The mean population density and centroid number by DA (N = 330) were 3090.2 (SD 2226.6) persons/km^2^ and 88.84 (SD 89.27) centroids, respectively.

**Table 1:** Mean BMI and distribution of observations by travel mode for categorical confounders (N = 4625)

	Mean BMI (SD)^a^	% Observations by travel mode			Total N
		Vehicular only	Mixed	Active only	
Total sample with BMI data	26.64 (5.13)	3587 (77.6%)	544 (11.8%)	494 (10.6%)	4625 (100%)
Transportation mode		–	–	–	
Vehicular transportation only	26.95 (5.16)				3587 (78.0%)
Mixed vehicular/active	25.79 (4.85)				544 (11.3%)
Active transportation only	25.32 (4.93)				494 (10.7%)
Individual variables					
Age					
19–34 years	25.19 (4.94)	68.2%	15.0%	16.8%	1238 (25.9%)
35–49 years	26.84 (5.13)	80.6%	10.7%	8.7%	1111 (24.0%)
50–64 years	27.23 (5.07)	80.8%	11.1%	8.1%	1474 (31.9%)
65 + years	27.54 (5.07)	81.8%	9.5%	8.7%	802 (17.3%)
Sex					
Male	27.4 (4.62)	78.4%	10.7%	10.9%	2247 (48.6%)
Female	25.92 (5.48)	76.8%	12.8%	10.4%	2378 (51.4%)
Physical activity					
< 3 days in prior week	27.5 (5.72)	85.8%	7.6%	6.6%	1759 (38%)
≥ 3 days in prior week	26.12 (4.66)	72.5%	14.3%	13.2%	2866 (62%)
Household variables					
Household income					
< $25,000	26.7 (6.02)	47.2%	17.9%	34.9%	195 (4.2%)
$25,000–$49,999	27.28 (5.45)	75%	9.7%	15.3%	639 (13.8%)
$50,000–$74,999	27.07 (5.33)	79.4%	10.4%	10.2%	943 (20.4%)
≥ $75,000	26.35 (4.9)	79.6%	12.3%	8.1%	2848 (61.6%)
Young children					
No children under 5	26.65 (5.14)	77.3%	11.8%	10.9%	4111 (88.9%)
Children under 5 in home	26.56 (5.05)	79.6%	11.3%	9.1%	514 (11.1%)
Neighbourhood variable					
Deprivation index					
Quintile 1 (most privileged)	26.27 (4.79)	84.8%	9.3%	5.9%	1345 (29.1%)
Quintile 2	26.22 (4.79)	79%	12.1%	8.9%	1048 (22.7%)
Quintile 3	26.59 (5.09)	72.6%	13%	14.4%	851 (18.4%)
Quintile 4	27.1 (5.1)	76.4%	10.2%	13.4%	675 (14.6%)
Quintile 5 (least privileged)	27.59 (6.1)	68.5%	15.9%	15.6%	706 (15.3%)

^a^SD is standard deviation

### Multi-level modeling

Results from Models 1–4 that include all confounders are presented in Table 2. DIC difference greater than 5 indicates significant and meaningful model improvement [40]. Models with spatial random effect terms (Models 2 and 4) have better model fit than their non-spatial counterparts (Models 1 and 3) based on DIC comparisons. We report the results based on spatial models.

**Table 2:** Effect estimates for confounders in the final adjusted model with and without interaction (N = 4625)

	Without interaction		With interaction	
	Mode 1: Non-spatial	Model 2: Spatial	Model 3: Non-spatial	Model 4: Spatial
Independent variables	Effect estimate (posterior mean, 95% CrI)		Effect estimate (posterior mean, 95% CrI)	
Individual variables				
Age^a^				
35–49 years	*0.065 (0.05, 0.079)*	*0.065 (0.05, 0.08)*	*0.065 (0.05, 0.079)*	*0.064 (0.05, 0.079)*
50–64 years	*0.091 (0.077, 0.105)*	*0.092 (0.078, 0.106)*	*0.092 (0.078, 0.106)*	*0.092 (0.078, 0.106)*
65 + years	*0.089 (0.071,0.106)*	*0.092 (0.074, 0.109)*	*0.09 (0.073, 0.108)*	*0.092 (0.074, 0.109)*
Sex^b^				
Female	*−* *0.068 (− 0.077, − 0.059)*	*− 0.068 (− 0.077, − 0.059)*	*− 0.067 (− 0.077, − 0.059)*	*− 0.068 (− 0.077, − 0.059)*
Physical activity^c^				
≥ 3 days in prior week	*− 0.036 (− 0.046, − 0.025)*	*− 0.035 (− 0.046, − 0.025)*	*− 0.036 (− 0.046, − 0.025)*	*− 0.035 (− 0.046, − 0.025)*
Travel mode^d^				
Mixed vehicular/active travel	*− 0.021 (− 0.037, − 0.005)*	*− 0.02 (− 0.036, − 0.004)*	0.001 (− 0.067, 0.075)	0.005 (− 0.058, 0.072)
Active travel only	*− 0.044 (− 0.062,− 0.027)*	*− 0.043 (− 0.06, − 0.025)*	0.005 (− 0.049, 0.061)	0.011 (− 0.04, 0.06)
Household variables				
Household income^e^				
$25,000–$49,999	− 0.016 (− 0.045, 0.014)	− 0.016 (− 0.046, 0.014)	0.002 (− 0.037, 0.044)	0.003 (− 0.032, 0.041)
$50,000–$74,999	− 0.026 (− 0.054, 0.003)	− 0.025 (− 0.054, 0.004)	− 0.006 (− 0.043, 0.035)	− 0.004 (− 0.038, 0.032)
≥ $75,000	*− 0.036 (− 0.063,− 0.008)*	*− 0.035 (− 0.063, − 0.007)*	− 0.014 (− 0.05, 0.026)	− 0.012 (− 0.043, 0.024)
*Transportation* mode * household income^f^	**–**			
Mixed vehicular/active travel				
$25,000–$49,999			0.016 (− 0.073, 0.097)	0.013 (− 0.067, 0.091)
$50,000–$74,999			− 0.017 (− 0.099,0.059)	− 0.02 (− 0.095, 0.052)
≥ $75,000			− 0.031 (− 0.107, 0.039)	− 0.035 (− 0.105, 0.031)
Active *transportation* only				
$25,000-$49,999			− 0.056 (− 0.124, 0.011)	− 0.059 (− 0.122, 0.005)
$50,000-$74,999			− 0.048 (− 0.114, 0.018)	− 0.052 (− 0.113, 0.009)
≥ $75,000			− 0.055 (− 0.115, 0.005)	*− 0.061 (− 0.115, − 0.007)*
Young children^g^				
Children under 5 in home	*0.029 (0.01,0.048)*	*0.027 (0.009, 0.046)*	*0.029 (0.01, 0.048)*	*0.027 (0.009, 0.046)*
Neighbourhood variables				
Deprivation index^h^				
Quintile 2	0.0001 (− 0.017,0.018)	− 0.003 (− 0.021, 0.014)	− 0.001 (− 0.018, 0.018)	− 0.003 (− 0.021, 0.015)
Quintile 3	*0.021 (0.003, 0.04)*	0.014 (− 0.006, 0.034)	*0.021 (0.003, 0.04)*	0.015 (− 0.005,0.034)
Quintile 4	*0.034 (0.014, 0.054)*	*0.028 (0.006, 0.048)*	*0.034 ( 0.014, 0.054)*	*0.028 (0.006, 0.049)*
Quintile 5 (least privileged)	*0.053 (0.033, 0.074)*	*0.042 (0.019, 0.065)*	*0.053 (0.033, 0.074)*	*0.043 (0.019, 0.065)*
Population density	− 0.001 (− 0.009, 0.006)	− 0.002 (− 0.009, 0.006)	− 0.001 (− 0.008, 0.006)	− 0.001 (− 0.009, 0.006)
Road centroids	− 0.004 (− 0.008, 0.001)	− 0.003 (− 0.007, 0.002)	− 0.004 (− 0.008, 0.001)	− 0.003 (− 0.007, 0.001)
Can-ALE	*− 0.006 (− 0.013, − 0.00,001)*	− 0.005 (− 0.012, 0.002)	*− 0.007 (− 0.013, − 0.001)*	− 0.005 (− 0.012, 0.002)
DIC	− 3498.21	− 3504.3	− 3495.43	− 3503.87

Italic indicates statistical significance (95% credible Interval does not cover zero)

*DIC* deviance information criterion. The lower DIC, the better the model fits the data

^a^Age 19–34 years is reference category

^b^Male is reference category

^c^< 3 days in prior week is reference category

^d^Vehicular travel only is reference category

^e^Income < $25,000 is reference category

^f^Vehicular travel only with household income < $25,000 is reference category

^g^No children under 5 is reference category

^h^Deprivation quintile 1 (most privileged) is reference category

In both models, age had a significant and positive association with BMI. Interestingly, after controlling for the effect of socio-demographic confounders on BMI, the presence of young children in the household became significant. This association could be due to lack of sleep [41] or more complex family child interactions [42, 43]. In contrast, females and participants who engaged in leisure time physical activity more than 3 days in the past week had lower BMI. Participants residing in DAs with the 4th and 5th quintile deprivation (least privileged) have higher BMI, while the other three urban forms, population density, the Can-ALE, and road centroids are not significantly associated with BMI.

In the main effects model (Model 2), significant and meaningfully lower BMI were observed for mixed (β = − 0.02; 95% CrI: − 0.036 to − 0.004) and active only (β = − 0.043; CrI: − 0.06 to − 0.025) compared to vehicular only travellers. Individuals in the highest income category had significantly lower BMIs than their counterparts in the lowest income category (β = − 0.035; CrI: − 0.063 to − 0.007). These significant associations however, were not found in the model with interaction terms between travel mode and household income (Model 4). Significantly lower BMI (β = − 0.061; CrI: − 0.115 to − 0.007) was observed for active only travellers in the highest income level (≥ $75,000) compared to active only travellers in the lowest income category (< $25,000).

## Discussion

The objectives of this study were to examine the association between travel mode and BMI among urban Canadians, and to assess the potential modifying effect of income on the relationship between travel mode and BMI. The SHTS data was linked to Census data for DA units in order to account for the “weight of place” [14] in our multilevel analysis. Our study is distinctive in two respects that address some of the limitations of past research. In particular, combining a trip diary with health measures is novel and permitted the simultaneous assessment of detailed travel mode and health information. The inclusion of random effects at the household level and spatially at the DA level allowed us to account for the heterogeneity between households and DAs.

Mode of travel was associated with adult BMI, even after controlling for participation in leisure time physical activities. Individuals who reported active only or mixed modes of travel had a significantly lower BMI than those who reported vehicular only travel, suggesting that active travel confers a health benefit. This finding mirrors that of Wen et al. [44], who report that Australian adults who commute to work by car are 1.13 (95% CI 1.01–1.27) times more likely to be overweight/obese than those who walk, cycle or use public transit. Evidence of a consistent relationship between active transportation and BMI is beginning to emerge; a recent longitudinal study noted a significant reduction in BMI among British adults who switched from private vehicular transport to active or public transport compared to participants with continued private vehicle use (− 0.32, 95% CI − 0.60 to − 0.05) [45].

The interaction between travel mode and household income was significantly associated with BMI, and offers insights concerning the disparate benefits of active transportation dependent on income. Mean BMI was significantly lower for active only travellers in the highest income category, but not in other lower income categories. This finding suggests that individuals of lower income may not benefit from the active transportation associated decreases in BMI observed at higher income categories. Interventions that increase active transportation may increase inequalities in BMI, particularly when an intervention is of greater benefit to advantaged than to disadvantaged groups [46]. Data on potential intervention-generated inequalities are lacking for many intervention types despite the observation that the efficacy of health interventions may be socially patterned [46, 47]. The results of this cross-sectional study advance the hypothesis that social position may limit the opportunity to benefit from active transportation. It is possible that active transportation use is insufficient to overcome the additional barriers to healthy weight maintenance imposed by compound disadvantage [47] among the poorest individuals. Interventions promoting active transportation to reduce BMI prevalence should therefore, parallelly take into account socioeconomic factors.

Deprivation was significantly associated with adult BMI at the DA Census unit level, such that individuals in the most deprived neighborhoods (quintiles 4 and 5) had significantly higher BMIs than their counterparts in the least deprived neighborhoods (quintile 1). The result is generally consistent with the existing literature and indicative of an area-level effect independent of individual sociodemographic characteristics and physical activity behaviors. A comparable study of Canadian adults reported an increased BMI score of 0.12 for each one-unit increase in neighborhood material deprivation [14]; an Australian analysis found that living in the most versus least disadvantaged area was associated with an average difference in BMI of 1.08 and 0.93 for women and men, respectively [48]. While an area’s socioeconomic status is associated with the body weight of its residents, experts caution against the wholesale application of the deprivation amplification concept to health policy [49]. Consideration should be given to the wider socioeconomic and cultural context, including the socioeconomic differences in motives and means for active transportation behavior [17, 49].

Our study emphasizes the importance of using a spatial model that accounts for unmeasured area-level confounders, which likely have spatial structures that could be associated with BMI. Different from non-spatial models (Models 1 and 3), the spatial models (Models 2 and 4), either with interaction terms or not, show that participants living in neighborhoods with the 3rd quintile deprivation do not have greater BMI compared with those living in the least deprived neighborhoods. This finding suggests that these participants might benefit from some spatially structured neighborhood factors, which are not accounted for in our analysis but mean participants had lower BMI. Using a spatial model also avoids this Type I error regarding the association between the Can-ALE and BMI. The non-spatial models (Models 1 and 3) incorrectly identify the Can-ALE as a negative contributor to BMI when it, in fact, is not. Likewise, the significance of the interaction between household income and travel modes (in Model 4 but not Model 3) indicates that high-income and vehicular-travel only participants would have lower BMI only when certain factors (missing in our analysis but represented with spatial random effects) are present in their neighborhoods.

Several limitations of this study are worth mentioning. The findings presented here are subject to various sources of bias that relate to both sample selection (given the voluntary nature of SHTS participation) and our reliance on self-reported data (given that individuals often underestimate BMI and/or overestimate physical activity). Because analysis was limited to individuals with weekday trip data, individuals who are unemployed or have mobility difficulties may be disproportionately excluded. The insignificance of urban form variables was unexpected given the literature linking physical activity and BMI to features of the built environment [33–35]. Rather than a true lack of association, this finding may reflect the size of our area-level unit or how these features were measured for this study. Also, given the deficiency in the 2011 Census data, the 2013 SHTS data was linked to the 2006 Census data. This mismatch might affect the final statistical results. A similar limitation is that the 2006, rather than 2011, Can-ALE was used in our analysis due to data unavailability. Finally, it is clear that obesity is a complex process that is influenced by many factors relating to diet quantity and quality [50]. Unfortunately, since participants’ eating behaviors were not collected in our survey, we were unable to account for nutritional information in the analysis. Future research could address these limitations by collecting daily travel behaviors over a longer time period and collecting eating behavior data.

## Conclusions

Strategies to increase active transportation use can support healthy weight maintenance, but the opportunity to benefit from active transportation use may be limited by low income or other markers of disadvantage. Policymakers should ensure that well-intentioned efforts to promote active transportation do not inadvertently reinforce social inequalities in BMI. Future research should use spatial statistical models, such as the one presented in this study, especially when contextual effects at the area-level are explored.

## Additional file

**Additional file 1. Appendices.**

## Abbreviations

BMI: body mass index
Can-ALE: Canadian active living environments
CI: 95% confidence interval
CrI: 95% credible interval
DA: dissemination area
DIC: deviance information criterion
INSPQ: Institut National de Santé Publique du Québec
SES: socioeconomic status
SHTS: Saskatoon Household Travel Survey

## Footnotes

1 We also fitted models with both non-spatial and spatial random effects, which are known as the convolution model. Compared with the models with spatial random effects only, results were almost identical and model fitting did not significantly improve. For parsimony reasons, we chose Models 2 and 4 as our final models.

## References

[1] Bassett MT, Perl S (2004). Obesity: the public health challenge of our time. Am J Public Health.

[2] Twells LK, Gregory DM, Reddigan J, Midodzi WK (2014). Current and predicted prevalence of obesity in Canada: a trend analysis. C Open.

[3] Guh DP, Zhang W, Bansback N, Amarsi Z, Birmingham CL, Anis AH (2009). The incidence of co-morbidities related to obesity and overweight: a systematic review and meta-analysis. BMC Public Health.

[4] Canadian Obesity Network. Report card on access to obesity treatment for adults in Canada, 2017. 2017.

[5] Warburton DER, Nicol CW, Bredin SSD (2006). Health benefits of physical activity: the evidence. CMAJ.

[6] Colley R, Garriguet D, Janssen I, Craig CL, Clarke J, Tremblay MS (2011). Physical activity of Canadian adults: accelerometer results from the 2007 to 2009 Canadian Health Measures Survey. Health Rep.

[7] Centers for Disease Control and Prevention. Transportation health impact assessment toolkit. 2011.

[8] Sallis JF, Frank LD, Saelens BE, Kraft MK (2004). Active transportation and physical activity: opportunities for collaboration on transportation and public health research. Transp Res Part A Policy Pract.

[9] Fox KR, Hillsdon M (2007). Physical activity and obesity. Obes Rev.

[10] Wanner M, Götschi T, Martin-Diener E, Kahlmeier S, Martin BW (2012). Active transport, physical activity, and body weight in adults a systematic review. Am J Prev Med.

[11] Saunders LE, Green JM, Petticrew MP, Steinbach R, Roberts H (2013). What are the health benefits of active travel? A systematic review of trials and cohort studies. PLoS One.

[12] Ross NA, Tremblay S, Khan S, Crouse D, Tremblay M, Berthelot JM (2007). Body mass index in urban Canada: neighborhood and metropolitan area effects. Am J Public Health.

[13] McLaren L (2007). Socioeconomic status and obesity. Epidemiol Rev.

[14] Matheson FI, Moineddin R, Glazier RH (2008). The weight of place: a multilevel analysis of gender, neighborhood material deprivation, and body mass index among Canadian adults. Soc Sci Med.

[15] McNeill LH, Kreuter MW, Subramanian SV (2006). Social environment and physical activity: a review of concepts and evidence. Soc Sci Med.

[16] Sallis JF, Glanz K (2009). Physical activity and food environments: solutions to the obesity epidemic. Milbank Q.

[17] Pampel FC, Krueger PM, Denney JT (2010). Socioeconomic disparities in health behaviors. Annu Rev Sociol.

[18] Alphonsus KB, Waldner C, Fuller D (2018). Examining the association between area level deprivation and vehicle collisions that result in injury. Can J Public Health.

[19] Morency P, Gauvin L, Plante C, Fournier M, Morency C (2012). Neighborhood social inequalities in road traffic injuries: the influence of traffic volume and road design. Am J Public Health.

[20] Fuller D, Winters M (2017). Income inequalities in bike score and bicycling to work in Canada. J Transp Health.

[21] Scott MM, Dubowitz T, Cohen DA (2009). Regional differences in walking frequency and BMI: What role does the built environment play for Blacks and Whites?. Health Place.

[22] Law J, Chan PW (2011). Monitoring residual spatial patterns using Bayesian hierarchical spatial modelling for exploring unknown risk factors. Trans GIS.

[23] City of Saskatoon Infrastructure Services Department. 2013 Household Travel Survey [Internet]. 2014 [cited 2016 Aug 26]. https://www.saskatoon.ca/sites/default/files/documents/transportation-utilities/transportation/planning/Attachment3 Technical Report HTS_FollowUp_report.pdf.

[24] Statistics Canada. Dissemination area (DA)—Census Dictionary [Internet]. 2012 [cited 2015 Sep 11]. p. 1. http://www12.statcan.gc.ca/census-recensement/2011/ref/dict/geo021-eng.cfm.

[25] Health Canada. Canadian community health survey, Cycle 2.2, Nutrition (2004) : a guide to accessing and interpreting the data [Internet]. Office of Nutrition Policy and Promotion, Health Canada; 2006. https://www.canada.ca/en/health-canada/services/food-nutrition/food-nutrition-surveillance/health-nutrition-surveys/canadian-community-health-survey-cchs/canadian-community-health-survey-cycle-2-2-nutrition-2004-guide-accessing-interpreting-data-health-ca.

[26] Centers for Disease Control and Prevention. About Child & Teen BMI [Internet]. 2015 [cited 2015 Sep 11]. https://www.cdc.gov/healthyweight/assessing/bmi/childrens_bmi/about_childrens_bmi.html#HowIsBMICalculated.

[27] Statistics Canada. Age: dictionary, census of population. 2016.

[28] Statistics Canada. Canadian community health survey—annual component. 2018.

[29] Employment and Social Development Canada. Indicators of well-being in Canada: financial security-Family income [Internet]. 2016 [cited 2016 Aug 26]. http://www.statcan.gc.ca/pub/89-503-x/2010001/article/11388-eng.htm.

[30] Milton K, Bull FC, Bauman A (2011). Reliability and validity testing of a single-item physical activity measure. Br J Sports Med..

[31] Pampalon R, Hamel D, Gamache P, Raymond G (2009). A deprivation index for healthy planning. Chronic Dis Can.

[32] Ross N, Wasfi R, Herrmann T, Gleckner W. Canadian active living environments database (Can-ALE): user manual & technical document [Internet]. 2018. http://canue.ca/wp-content/uploads/2018/03/CanALE_UserGuide.pdf. Accessed 12 Dec 2018.

[33] Saelens BE, Sallis JF, Black JB, Chen D (2003). Neighborhood-based differences in physical activity: an environment scale evaluation. Am J Public Health.

[34] Frank LD, Andresen MA, Schmid TL (2004). Obesity relationships with community design, physical activity, and time spent in cars. Am J Prev Med.

[35] Ewing R, Schmid T, Killingsworth R, Zlot A, Raudenbush S, Marzluff JM, Shulenberger E, Endlicher W, Alberti M, Bradley G, Ryan C, Simon U (2008). Relationship between urban sprawl and physical activity, obesity, and morbidity. Urban Ecol An Int Perspect Interact Between Humans Nat.

[36] Was RA, Dasgupta K, Eluru N, Ross NA (2016). Exposure to walkable neighbourhoods in urban areas increases utilitarian walking: longitudinal study of Canadians. J Transp Health.

[37] Lunn DJ, Thomas A, Best N, Spiegelhalter D (2000). WinBUGS—a Bayesian modelling framework: concepts, structure, and extensibility. Stat Comput.

[38] Haining R, Law J, Griffith D (2009). Modelling small area counts in the presence of overdispersion and spatial autocorrelation. Comput Stat Data Anal.

[39] Duncan EW, White NM, Mengersen K (2017). Spatial smoothing in Bayesian models: a comparison of weights matrix specifications and their impact on inference. Int J Health Geogr.

[40] Lunn D, Jackson C, Best N, Thomas A, Spiegelhalter D (2012). The BUGS book: a practical introduction to bayesian analysis.

[41] Cappuccio FP, Taggart FM, Kandala N-B, Currie A, Peile E, Stranges S (2008). Meta-analysis of short sleep duration and obesity in children and adults. Sleep.

[42] Edvardsson K, Lindkvist M, Eurenius E, Mogren I, Small R, Ivarsson A (2013). A population-based study of overweight and obesity in expectant parents: socio-demographic patterns and within-couple associations. BMC Public Health.

[43] Lindkvist M, Ivarsson A, Silfverdal SA, Eurenius E (2015). Associations between toddlers’ and parents’ BMI, in relation to family socio-demography: a cross-sectional study. BMC Public Health.

[44] Wen LM, Orr N, Millett C, Rissel C (2006). Driving to work and overweight and obesity: findings from the 2003 New South Wales Health Survey, Australia. Int J Obes.

[45] Martin A, Panter J, Suhrcke M, Ogilvie D (2015). Impact of changes in mode of travel to work on changes in body mass index: evidence from the British Household Panel Survey. J Epidemiol Community Health.

[46] Lorenc T, Petticrew M, Welch V, Tugwell P (2013). What types of interventions generate inequalities? Evidence from systematic reviews: Table 1. J Epidemiol Community Health.

[47] White M, Adams J, Heywood P, Babones SJ (2009). How and why do interventions that increase health overall widen inequalities within populations?. Social inequality and public health.

[48] King T, Kavanagh AM, Jolley D, Turrell G, Crawford D (2006). Weight and place: a multilevel cross-sectional survey of area-level social disadvantage and overweight/obesity in Australia. Int J Obes.

[49] Macintyre S (2007). Deprivation amplification revisited; or, is it always true that poorer places have poorer access to resources for healthy diets and physical activity?. Int J Behav Nutr Phys Act.

[50] Ludwig DS, Ebbeling CB (2018). The carbohydrate-insulin model of obesity beyond “calories in, calories out”. JAMA Int Med.

