# Supplementary material for: Household income, active travel, and their interacting impact on body mass index in a sample of urban Canadians: a Bayesian spatial analysis
Source: Int J Health Geogr. 2019 Feb 6;18:4. doi: 10.1186/s12942-019-0168-x (PMC6366056; doi:10.1186/s12942-019-0168-x)
Supplement: Supplementary file 1 — Additional file 1. Appendices. [file 12942_2019_168_MOESM1_ESM.docx]

**Appendix 1: Bivariate regression between BMI and confounders**

Bivariate regression was performed between BMI and individual characteristics. Continuous variables which failed to meet the linearity assumption were categorized into two or more categories. In particular, participants who are male, 19-34 years old, travel with vehicles only, and live in the most deprived DAs are the reference groups in the analysis. The results of the bivariate analyses with BMI are presented in Table A1. With the exception of household income, the presence of young children, deprivation quintiles 2 and 3, population density, and Can-ALE, the other confounders are significant at the p-value<0.01 level.

**Table A1.** Bivariate regression for independent variables

| Candidate confounders | Effect estimate | p-value |
| --- | --- | --- |
| Individual variables |  |  |
| Age^a^ |  |  |
| 35-49 years | **0.064** | **<0.001** |
| 50-64 years | **0.079** | **<0.001** |
| 65+ years | **0.091** | **<0.001** |
| Sex^b^ | **-0.062** | **<0.001** |
| Physical activity^c^ | **-0.046** | **<0.001** |
| Travel mode^c^ |  |  |
| *Mixed vehicular/active travel* | **-0.043** | **<0.001** |
| *Active travel only* | **-0.062** | **<0.001** |
| Household variables |  |  |
| Household income^d^  *$25000-$49999*  *$50000-$74999*  *>$75000* | 0.026  0.019  -0.006 | 0.08  0.19  0.67 |
| Young children^e^ | -0.002 | 0.78 |
| Neighbourhood variables |  |  |
| Deprivation index^f^  *Quintile 2*  *Quintile 3*  *Quintile 4*  *Quintile 5* | -0.002  0.011  **0.03**  **0.042** | 0.83  0.19  **<0.001**  **<0.001** |
| Population density | 0.001 | 0.61 |
| Road centroids | **-0.003** | **0.008** |
| Can-ALE | 0.002 | 0.45 |

^a^ Age 19-34 years is reference category

^b^Male is reference category

^c^<3 days in prior week is reference category

^d^Household income <$25000 is reference category

^e^No children under 5 is reference category

^f^Deprivation quintile 1 (most privileged) is reference category

**Appendix 2: Prior specification, model implementation, and WinBUGS code**

Without genuine prior knowledge, we specified a non-informative prior Uniform(-∞, +∞) to the intercept α. A vague prior, Uniform(0,100), was assigned for the standard deviation of the individual-level, household-level, and DA-level non-spatial random effects (represented as sd.u1, sd.u2, and sd.u3, respectively, in the WinBUGS code below). For the spatial random effect, s3, we used an intrinsic Conditional Autoregressive (iCAR) distribution that accounts for spatial structures in the data that have not been explained by DA-level confounders. Similarly, the prior for the standard deviation of the spatial random effect (sd.s3) was Uniform(0,100). A prior of a normal distribution with mean zero and a large variance, 10,000, was specified for the coefficients.

All four Bayesian models were implemented in WinBUGS 1.4. For each model, two parallel chains with diverging initial values were fitted. Model convergence was visually examined by the history plot, trace plot, and Gelman-Rubin plot. The initial 10,000 iterations, where the models converged, were discarded as burn-ins. We ran another 30,000 iterations for each chain, resulting in a total number of 60,000 samples for the posterior inference. Sensitivity analysis of priors (by specifying different vague priors to unknown parameters) showed that the results are not sensitive to prior specifications. The WinBUGS code for the spatial model with interaction terms (Model 4) is provided below.
